# Mechanisms of tertiary lymphoid structure formation: cooperation between inflammation and antigenicity

**DOI:** 10.3389/fimmu.2023.1267654

**Published:** 2023-09-21

**Authors:** Shrijan Khanal, Andreas Wieland, Andrew J. Gunderson

**Affiliations:** ^1^ Division of Surgical Oncology, Comprehensive Cancer Center, The Ohio State University, Columbus, OH, United States; ^2^ Department of Otolaryngology, Comprehensive Cancer Center, The Ohio State University, Columbus, OH, United States

**Keywords:** B cell, tumor immunity and immunotherapy, tertiary lymphoid structures, adaptive immunity, T cell, T cell - B cell collaboration

## Abstract

To mount an effective anti-tumor immune response capable of controlling or eliminating disease, sufficient numbers of lymphocytes must be recruited to malignant tissue and allowed to sustain their effector functions. Indeed, higher infiltration of T and B cells in tumor tissue, often referred to as “hot tumors”, is prognostic for patient survival and predictive of response to immunotherapy in almost all cancer types. The organization of tertiary lymphoid structures (TLS) in solid tumors is a unique example of a hot tumor whereby T and B lymphocytes aggregate with antigen presenting cells and high endothelial venules reflecting the cellular organization observed in lymphoid tissue. Many groups have reported that the presence of preexisting TLS in tumors is associated with a superior adaptive immune response, response to immunotherapy, and improved survivorship over those without TLS. Accordingly, there is significant interest into understanding the mechanisms of how and why TLS organize so that they can be elicited therapeutically in patients with few or no TLS. Unfortunately, the most commonly used mouse models of cancer do not spontaneously form TLS, thus significantly restricting our understanding of TLS biology. This brief review will summarize our current state of knowledge of TLS neogenesis and address the current gaps in the field.

## Introduction

Given the broad clinical success of immunotherapies in cancer patients, the intense focus on tumor immunology research in this century is warranted. However, most cancer patients still do not respond to current immunotherapy options necessitating a contextual understanding of the biomarkers that predict response and how to achieve optimal anti-tumor immunity. To date, most tumor immunology research has focused on how T cells and macrophages function within the tumor microenvironment, attributed to their relative abundance in tumors and their importance in mediating an effector response. However, renewed attention is being given to other tumor infiltrating immune subsets, including the roles and functions of B cells (1, 2). This in part due to the presence of spontaneously derived tertiary lymphoid structures (TLS), which are aggregations of T and B cells apportioned around antigen presenting cells and high endothelial venules (HEV); organized follicles only found in homeostatic conditions in secondary lymphoid organs (SLO) (3, 4). TLS are not only prognostic for survival in cancer patients (5, 6), but also predictive of response to immune checkpoint blockade in certain cancer types (7–12). The question remains, however, why tumors in some patients contain TLS while others do not. These answers will reveal how to therapeutically target these pathways to elicit TLS formation *de novo*. Here, we curate and summarize what is currently known about the cellular and molecular determinants of TLS neogenesis in solid tumors.

### Defining tertiary lymphoid structures

TLS are B and T cell lymphoid aggregates observed histologically in chronically inflamed peripheral tissues such as those found in solid tumors, persistent infections, transplanted organs, and many autoimmune pathologies (6). Reports on the tissue location of TLS varies and has caused some confusion about what is a bonafide TLS. Although it remains theoretically possible that an independent TLS could develop within a primary (bone marrow, thymus) or SLO (lymph node, spleen, tonsil), due to their histological similarities there is not yet a reliable way to differentiate between a newly formed TLS and the residual lymphoid tissue that precedes it. Lymphocytes comprise the bulk of the immuno-cellular aggregate, but other prototypical features include CD21^+^ follicular dendritic cells (FDC) and PNAd^+^ HEV. TLS have been observed in almost every solid tumor type and correlate with improved survival in cancer patients (13), although contrary outcomes for HCC patients have been reported (14). TLS can also arise in metastatic lesions and associate with longer survival (15). Furthermore, a subset of TLS^+^ patients exhibit germinal center formation directly in tumor tissue whereby BCL6-expressing B cells are rapidly dividing within a segregated CD20^+^ B cell zone marked by CD21^+^CD23^+^ follicular dendritic cells and marginated by T cells (**Table 1**). These “mature-TLS” are considered by some investigators to be the only representative version of TLS, and indeed patient tumors with *in situ* germinal center reactions have the longest survival rates (16, 17). Moreover, some groups have reported that the determining factor in TLS as a prognostic biomarker is the intratumoral location and density of these aggregates (18, 19). The extended survivorship in TLS^+^ patients also correlate with the substantial increases in the anti-tumor immune phenotype including higher lymphocyte densities (15), production of tumor-reactive antibodies (20, 21), increased memory T and B cells (16), increased mature dendritic cells (22), plasma cell differentiation (23, 24) and T-helper type 1 responses (22). Although we do not yet know the precise effector mechanisms of how TLS function to enhance anti-tumor immunity, the prevailing hypothesis is that TLS sustain a proximal niche for more frequent interactions between antigen presenting cells and lymphocytes, thereby facilitating an improved immune phenotype *in situ*. More specifically, perhaps the predominant contribution provided by TLS, especially those with germinal centers, is improved humoral immunity where antibody secreting cells, are locally differentiated from TLS-resident B cell precursors (21, 23, 24). In turn, these newly produced antibodies may directly target tumor cells for antibody dependent cell mediated cytotoxicity (20, 23), activate the complement pathway (25), and/or enhance antigen uptake and cross-presentation to T cells (26). Furthermore, activated B cells are extremely efficient antigen presenting cells (27), a function previously shown to shape CD4^+^ T cell responses in human tumors (28). Regardless of exactly how TLS support anti-tumor immunity, the rationale to understand the mechanisms of TLS formation in patients for improved clinical benefit is clear.

**Table 1:** Comparison of spatial lymphocyte patterning in human tumors.

Structure type	Cell types	Organization/Size	HEV	Markers	Prognosis
Mature-TLS	CD4^+^ T, CD8^+^ T, B cell, FDC, plasma cells, reticular cells	High/large-strict T and B cell zones like germinal center, thousands of clustered cells	Yes	CD20, CD3, CD8, CD21, CD23, PNAd, CD208, CD79A, CD138, BCL6, Ki67	+++
Early/Immature-TLS	CD4^+^ T, CD8^+^ T, B cell	Moderate/medium-lymphocytes clustering but no specific zones	Maybe	CD20, CD3, CD79A, CD8, PNAd	++
Lymphocyte aggregate	T cell, B cell, macrophage	Low/small – loose aggregate, can be comprised of only T cells or only B cells	No	CD20, CD3, CD68	+

Diversity of lymphoid aggregate types observed in human tumors. The cell types most commonly observed within the aggregate type is listed along with the markers used to identify those cells types, morphological features and associated with patient outcomes relative to tumors without an aggregate. +++: longest overall survival; ++: moderate improvement in overall survival; +: small improvement in overall survival.

### Mouse models of tumor TLS formation

Unfortunately, tumor models derived from syngeneic cell lines implanted in the subcutaneous compartment of mice do not exhibit naturally formed TLS, preventing the interrogation of TLS development through the usual genetic and cellular experimental approaches. However, an increasing number of groups have reported the existence of intratumoral TLS in specific contexts and the information generated from these studies has offered us some insight into the regulators of TLS development. Joshi et al. reported the presence of TLS in a transgenic (*Kras* ^Lox-STOP-Lox(LSL)-G12D^ *Trp53* ^flox/flox^) mouse model of lung adenocarcinoma that reflect TLS observed in human lung adenocarcinoma patients (25). Rodriguez et al. reported that antigen strength is linked with quantity and quality of TLS; but also as a result of factors in the tumor microenvironment in specific anatomical locations. They observed spontaneous TLS formation in B16-OVA tumors injected intraperitoneally (I.P.) but not subcutaneously (S.C.) (26) or with the parental B16 cell line. Similarly, Ng et al. published that only highly mutated mouse lung cancer cell lines could induce TLS formation, due to the increased tumor immunogenicity (29). Thus, the antigen strength and the site of injection might also affect TLS formation in tumors. Schrama et al. observed that lymphotoxin-α (LT-α) expression is effective in eradicating subcutaneous B16 melanoma tumors associated with the induction of peripheral lymphoid neogenesis (27). Delvecchio et al. reported that TLSs do not form ubiquitously in the transgenic “KPC” (*Kras* ^G12D^, *p53* ^R172H^, *Pdx-1-Cre*) pancreatic cancer model and that injecting chemokines (CXCL13/CCL21) intratumorally in the orthotopic model of PDAC elicits TLS formation (28). Finally, Johansson-Percival reported that in spontaneous pancreatic neuroendocrine tumors (insulinomas) arising in RIP1-Tag5 mice, treatment with LIGHT-vascular targeting peptide (VTP) fusion compound normalizes the vasculature of the tumor leading to TLS-neogenesis in these neuroendocrine tumors (29). To dissect the mechanism of TLS neogenesis more clearly, it will be imperative to expand the number of preclinical models that faithfully reflect TLS-immunobiology in cancer patients.

### Fibroblasts as key lymphoid tissue organizer cells

During lymphoid tissue ontogeny and organogenesis, lymphoid tissue inducer (LTi) and lymphoid tissue organizer (LTO) cells collaborate to recruit lymphocytes to an expanding follicular region and provide the initiating instructions for the compartmentalization of these follicles (30). Moreover, FDC, which do not have hematopoietic origin, are critical to maintaining the unique spatial organization of lymphoid follicles, primarily via CXCL13 secretion. However, LTi, LTO, and FDC cells do not exist in peripheral non-lymphoid tissue under homeostatic conditions and thus must either be recruited or co-opted from other sources to perform a similar function for TLS neogenesis. During embryogenesis, LTi cells are classified as a subset of innate lymphocytes (ILC) (bone marrow-derived in adults) (31) and initiate the LN anlagen between E12.5 and E15.5 in mice (gestational week 12-17 in humans) (32). Various cell types have been demonstrated to perform this function for tumor TLS including CD8^+^ T cells and NK cells (33, 34), CD4^+^ Th17 cells (35), B cells (36), and M1 polarized macrophages (37). Expression of lymphotoxin-α1β2 on LTi leads to upregulation of critical chemokines (CCL19, CCL21, CXCL12, and CXCL13) from lymphotoxin beta receptor (LTBR)-expressing stromal LTO cells which not only reinforces chemoattraction and retention of LTi cells but also mature T and B cells (30). These new emigrants form a fibroblastic reticular network around the activated fibroblasts serving as the cellular backbone for follicular development in the SLO. In tumors, an elegant study with B16-derived mouse melanomas revealed that cancer-associated fibroblasts (CAF - fibroblasts in tumors) obtain the key phenotypic features of LTO cells to recruit T and B cells for initial TLS-aggregation (34). Interestingly, only B16 tumors expressing ovalbumin (OVA) implanted intraperitoneally, but not subcutaneously, could generate TLS. Furthermore, CAF from B16-OVA tumors implanted intraperitoneally co-expressed VCAM1, ICAM1, and LTBR, distinct from subcutaneous CAF which were predominately VCAM1^-^ICAM^+^FAP^+^LTBR^low34^. Like in lymph node organogenesis (38–40), tumor necrosis factor receptor (TNFR) and LTBR signaling in CAF cooperated to promote TLS but in distinct ways. While TNFR signaling was required for the initial clustering of fibroblastic reticular networks by upregulating the B cell specific factors CXCL13, BAFF, and APRIL, LTBR signaling promoted expansion of the TLS through further production of chemokines (34). In this tumor model, CD8^+^ T cells and B cells collaborated to serve non-overlapping LTi functions, expressing TNFR1/2 and LTBR ligands respectively. Similarly, in a mouse model of the autoimmune condition, Sjogren’s syndrome, immune-stimulating fibroblasts in the salivary gland also promoted TLS formation, although primarily through IL-13, IL-22 and lymphotoxin (41). Once the reticular network has established the T and B cell aggregate, FDC are responsible for retaining that architecture and appear to derive from perivascular precursor cells under LTBR-regulated transdifferentiation (42). The characterization of tissue site-specific CAF phenotypes capable of TLS-organizing properties is an interesting concept and may help explain why induction of TLS in subcutaneous tumors is so challenging. It should be noted, however, that overexpression of LTBR ligands in tumor cells can overcome these microenvironmental deficits (43). Taken together, the data indicate that tumors with higher degrees of T cell inflammation, which can serve as LTi cells, create tumor microenvironments more permissive to TLS formation. Indeed, therapeutic strategies that promote T cell activation or recruitment, such as anti-PD1/PDL1 antibodies or cancer vaccines, can significantly boost TLS formation (33, 44–47). Despite this, there are contexts in which intratumoral T cell inflammation is high, but TLS formation remains absent (48, 49). Hence, infiltration of LTi cells in tumors is necessary but not sufficient to induce TLS and likely also requires programming of a specific LTO (CAF) phenotype.

### High endothelial venules control lymphocyte infiltration

Like lymph node endothelium, TLS^+^ tumors contain an increased proportion of blood vessels comprised of a unique phenotype of endothelial cell (EC) called a high endothelial venule (HEV). These vessels are identified by their expression of sialomucins, also known as peripheral node addressins (PNAd), and can be detected with the antibody clone MECA-79 that recognizes the carbohydrate epitope 6-sulpho sialyl Lewis-X (50, 51). The functional importance of this post-translational modification is that it is recognized by L-selectin (CD62L) which is expressed by naïve and memory T and B cells, allowing arrest on the HEV (52–54). Many groups have reported the clear association with HEV density in human tumors, lymphocyte infiltration and improved patient outcomes (55, 56). Accordingly, promotion of the HEV phenotype is a critical step preceding lymphocyte extravasation and follicular organization in tissue. This can be challenging given the known dysregulated endothelium commonly observed in tumors (57). To overcome these defects, work from three groups has revealed that agonizing the LTBR pathway, with or without VEGFR inhibition, while also blocking T cell checkpoints, promoted HEV differentiation from existing endothelial cells (33, 37, 47). Correspondingly, T cell infiltration of tumors was significantly improved, enriched for PD1^+^TCF1^+^ “stem-like” T cells which are the progenitors of exhausted T cells and necessary for response to immune checkpoint blockade therapies (58–60). Other groups have shown that stem-like T cells in tumors preferentially reside in TLS and lymphocyte clusters (61), dependent on interactions between CD62L and PNAd^+^ HEV (47), and can be reactive to tumor antigens (62). Like the LTO functions of CAF, the molecular determinants of endothelial cell conversion in HEV are dependent on TNFR and LTBR signaling. While TNFR signaling in EC appears to control spontaneous HEV acquisition of an immature status, LTBR signaling promotes functional maturation and maintenance of HEV. Activation of LTBR on EC leads to upregulation of the carbohydrate sulfotransferase, CHST4, and CCL19, CCL21, CXCL12, CXCL13 (63, 64), and EC-conditional LTBR KO mice have greatly impaired HEV formation (65). Interestingly, EC metaplasia to HEV in tumors may not be permanent but requires constitutive LTBR agonism (33). The TNFR ligands, TNFα and LTα and the LTBR ligands, LTα1β2 and LIGHT, can be expressed by dendritic cells (66–68), B cells (67, 69), NK cells and T cells (33, 34, 70) indicating that a threshold for immune cell trafficking must be overcome to maintain a self-amplifying loop in the therapeutic setting. HEV are also encapsulated by mural and fibroblastic reticular cells in the perivascular space, highlighting a functional cooperation with CAF in creating a hospitable region for TLS formation (52).

### Immunosuppression restricts TLS formation

Thus far, we have mostly reviewed the positive signals that regulate TLS formation but given the well documented immunosuppressive mechanisms that restrict immunity in tumors, it is likely TLS formation and activity is hindered similarly by these pathways. Less is known about the negative regulators of TLS, but independent groups have reported that CD4^+^ T-regulatory cells (Tregs) are suppressive of HEV formation and TLS activity (71–73). Joshi et al. first reported that selective depletion of Tregs liberated T cell activation in tumors from transgenic mouse lung cancer models. Both CD8^+^ T cells and CD4^+^ helper T cells increased proliferation rates in the absence of Tregs leading to greater TLS expansion. Interestingly, Treg residency in lung tumors appeared to concentrate in TLS regions vs. the rest of the surrounding tumor (72). Colbeck et al. published that Treg depletion in mice bearing carcinogen-induced sarcomas lead to HEV formation in tumors with no discernible impact on LN HEV formation (71). Lymphocyte infiltration into tumors was strongly correlative with HEV formation and reduced tumor growth. Interestingly, these phenotypes were dependent on TNFR signaling but not LTBR signaling, and CD11c^+^ cells were dispensible (71). One caveat to these studies is that Tregs were depleted systemically by the administration of diphtheria toxin in Foxp3^DTR^ mice precluding the interpretation that local Treg function within or near tumor-TLS was responsible for these results. Indeed, tumor-bearing animals succumbed to an autoimmune condition 2-3 weeks after Treg depletion (72). Finally, a study by Chaurio and colleagues demonstrated spontaneous TLS formation in intraperitoneal ovarian tumors implanted in CD4Cre-SATB1^flox/flox^ mice (73). These mice have a conditional deletion of the epigenetic modifier SATB1 in CD4^+^ T cells which results in a substantial shift in T-helper cell polarization from Tregs towards T-follicular helper cells (Tfh) (73). Tfh are critically important for B cell activation, specifically functioning in LN follicles and germinal centers by promoting B cell proliferation, survival, class switching, and affinity maturation (74–76). In multiple tumor studies, they have been shown to foster anti-tumor immunity (77–79). Hence, the absence of Tregs and the large increase in Tfh in these models is a natural prerequisite towards the development of TLS. Interestingly, TLS formation was greater in pancreatic cancer patients depleted of Tregs by low-dose cyclophosphamide in combination with whole tumor cell vaccination compared to vaccine alone, and Treg-low TLS were associated with longer survival in anti-PD1 treated sarcoma patients (10, 45, 46). Taken together, these studies point to the conclusion that blocking Treg activity is an important therapeutic strategy for promoting TLS formation.

### Contributions of antigens to TLS formation and activity

TLS are often associated with more vigorous anti-tumor immune responses and are hypothesized to directly contribute to their maintenance. However, the antigen specificities of the lymphocytes residing within TLS have not been determined to date. Hence, whether TLS or to what degree TLS are composed of tumor-reactive T and B cells is not known. A major technical challenge for the study of tumor-reactive B cells is their recognition of linear and conformational epitopes without predictable patterns such as HLA restriction for T cells, rendering the assessment of their antigen specificity cumbersome. While the study of tumor reactivity of intratumoral B cells might still be in its infancy, several studies have already demonstrated the presence of tumor- and auto-reactive B cells in human tumors (20, 21, 29, 80). The antigens targeted by these intratumoral B cells ranged from tumor-associated viral proteins (80) and endogenous retrovirus (ERV) proteins (29) to self-antigens (20, 21). In addition to the presence of B cells specific for tumor-associated HPV antigens in HPV+ head and neck cancer tumors (80), these tumors also contain substantial numbers of CD8^+^ T cells reactive against the same HPV antigens with a subset of cells phenotypically and functionally resembling “stem-like” T cells identified in preclinical mouse models (62). These data point towards a concerted interplay between tumor-reactive B and T cell responses which is further supported by a study linking increased tumor mutational burden, B cell and Tfh cell responses with improved responses to immune checkpoint inhibition in murine breast cancer models (79), the increased presence of conventional CD4^+^ T cell subsets clustering with germinal center B cells in HPV+ HNSCC (81), and a study demonstrating that germinal center formation in TLS of PDAC patients is associated not with overall TMB but with the presence of predicted MHC-I-restricted neoantigens (16). Of note, a novel lung adenocarcinoma mouse model allowing the detailed study of anti-tumor responses using B and T cell model antigens demonstrated the collaboration of tumor-specific B and Tfh cells to promote anti-tumor CD8+ T cell responses (78). Overall, these studies highlight the importance of studying tumor-specific immune responses, especially in terms of their contribution to TLS formation and activity.

## Discussion

**Figure 1:**
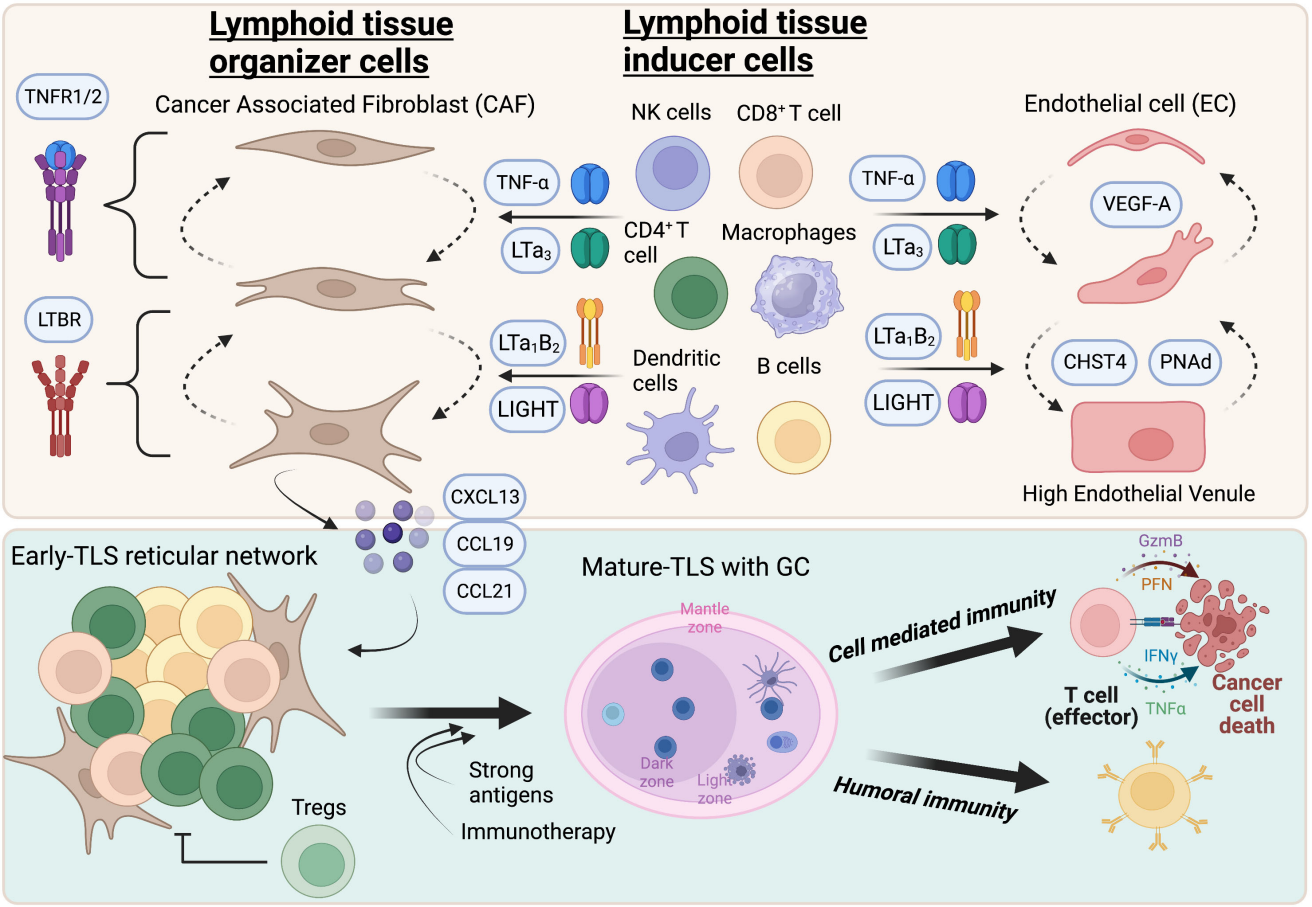
Highlights of TLS formation in tumors. TNFR and LTBR signaling in cancer associated fibroblasts and endothelial cells promote acquisition of LTO and HEV phenotypes respectively. While TNFR primes a reversible immature state, LTBR agonism promotes maturation and stability of TLS-inducing activity. The lymphocyte chemokines CXCL13, CCL19, and CCL21 are expressed by HEV and CAF that recruit new lymphocytes to form a reticular network that eventually manifests as a B and T cell aggregate, or “early”-TLS. Putative LTi cells that express TNFR and LTBR ligands include CD8+ T cells, CD4+ T cells, NK cells, B cells, dendritic cells and macrophages. Tumors that express strong antigens or those that respond to T cell activating immunotherapies, such as immune checkpoint inhibitors or vaccines, can contain germinal centers or “mature”-TLS. These patients have significant increases in both effector arms of the anti-tumor immune phenotype which leads to long-term tumor control and survival. *Created with BioRender.com*.

More questions than answers remain on the role of TLS in cancer immunity. This mini-review focuses primarily on how TLS form (**Figure 1**), but the data from these studies are clearly relevant to all aspects of TLS functionality in cancer and beyond. We have made good progress in characterizing TLS in human cancer but to address these questions more adequately, the expansion and accessibility of mouse tumor models where TLS develop is of critical importance. An important and still outstanding question in the field is the lack of evidence that tumor-reactive T and B cells reside directly within TLS, which is only presumed at this point. After residing in the TLS, where do these lymphocyte clones migrate to and what differentiation phenotypes do they acquire? The use of TCR and BCR transgenic mice and spatial transcriptomics are obvious tools that will help elucidate these points. Other future areas of exploration include the influence of the microbiome on TLS formation (82), how to predict the presence of TLS preoperatively from diagnostic tumor specimens with limited tissue amounts, and how cytotoxic therapies impact TLS (83, 84). What is clear, however, is that the standard immunology principles of inflammation and antigenic strength conspire to coordinate an organized TLS-immune response. Greater insights into this cooperation and how to therapeutically leverage these immunological relationships are needed to fully harness the potential of TLS in the clinic for all cancer patients.

## Author contributions

SK: Writing – original draft, Writing – review & editing. AW: Writing – original draft, Writing – review & editing, Visualization. AG: Visualization, Writing – original draft, Writing – review & editing, Conceptualization, Funding acquisition, Investigation, Supervision.
